# Indents

**Published:** 1918-08

**Authors:** 


### INDENTS

**☞Brief Articles of Technical or Scientific Value will receive notice and comment. Contributions invited.**

---

*Let Your Face So Shine* Out in Virginia City, Nev., there is an ancient Chinese storekeeper, Chung Kee by name, who for forty celestial years has been one of Nevada's notably happy men. Good days and bad, you will find him sitting at his receipt of custom, and smiling on all comers like some time-worn but kindly Buddha. He has made little money. Or what he has made he has given away again, or lost to debtors who wouldn't pay. And never has anyone been able to persuade him to get out an attachment for even the worst of them. Evidently he had some sort of philosophy. But just what was it, people wondered. And when, a few months ago, the town was to say good-by, with the proper ceremonies, to the thirteen young men of its first draft—who were also, almost all of them, in old Chung's books—people wondered, too, what would he do about that?

This is what he did: When, in his turn he walked down the line to say good-by, for each of the thirteen he was hiding a $5 gold piece in his hand. And later he explained.

"Why I do that? They alla samee good boy. They no want hurt me. I not goin' hurt them. All my life I neveh hurt nobody. That why, alla samee my face she shine. Bad fella', always you know him. Why? Alla samee his face she don't shine. I'm prett' old man. Prett' soon I gotta go die. An' I want my face be alla samee shinin' when I'm dead."

There you have it. You can make money unfairly out of war if you want to. It's always been done. But next time you do, go and take a look at yourself in the mirror, and see if you can find the shine. And while you are making your blood money, there are those dying today in Flanders and Picardy—yes; and those who are only able to fight the good fight here in America—whose faces are going to shine forevermore.

*Poor Kaiser; He Forgot the Yam*

Consider the Yankee yam. It's another of the many things the houses of Hapsburg and Hohenzollern forgot when they figured on catching all free Europe, having it toasted for dinner, and finishing off the meal with the United States as a dessert, Latin America as a cordial, and the Orient as an after-dinner smoke.

The American yam, or sweet potato, tnanks to the U-boat attempts on the world's food cargoes, has suddenly found recognition as a highly important food product. How extensively it might be used only the Teuton starvation campaign made us realize. The yam is easily cultivated. It takes little work. It stands the test of the dietitian.

Prepared as flour, it is one of the best substitutes for wheat. Six distinct kinds of yam flour are produced. Two of these are choice breakfast foods, sweet enough to serve without sugar. One is a starch flour, suitable for puddings, blancmanges, gravies, and the like—a rival to the cornstarch made from maize.

With the starch removed, the pulp makes pies and puddings. It resembles shredded cocoanut. The yam can be tinned or dried easily. It is very compact in its various manufactured and preserved forms, and can be readily transported. Its preparation for eating is simple, and even the peelings can be utilized commercially.

The yam has even more to say for itself. It contains sugar. In combination with peanuts, cowpeas, and beans, to afford the proteid and fat element, the yam can supply a well balanced and reasonably complete diet in which meats and the like need not be extensively included. More than eighty different food products can be made from this root, always a staple article of food in the southern states of America.

Poor Kaiser! All the things that he forgot—the spirit of free peoples, the rights of man, conscience, God, even the humble yam—rise up to fight against him.

*Enough Army Dentists* The dental requirements of an army of more than 5,000,000 men can now be met by the present force of the dental corps of the American army. Examinations for dental officers have been closed, and no further additions will be made to the corps for at least six months. At the time the United States declared a state of war to exist between this country and Germany the total number of dental officers was fifty-eight. The present force numbers 5,810. Commissions were offered to 5,467 dentists in all parts of the country, and all but 271 were accepted, a percentage of 95.1. A school for dental instruction has been started at Fort Oglethorpe, Ga. Eighty-five dental officers are assigned each month to take the two months' course. The first month is given over to 180 hours of general military instruction and training and the second to 70 hours' special military training and 100 hours devoted to professional subjects that have a definite relation to general practice of dentistry as it should be conducted in the army. Special dental infirmaries have been established in the camps and cantonments in this country to which every newly inducted soldier is sent for examination shortly after arrival in camp. In those cases where men need attention it is given. The average number of tooth fillings is from 225,000 to 250,000 a month. This figure does not include the examinations, treatments, extraction, or crown, bridge, and plate work. The practice followed in the camps is to first give treatment to those cases that require immediate attention. Special attention is paid in determining the number of chronic infections associated with teeth. It has been found that a number of diseases incapacitating men for military service are traceable to chronic infections about the teeth. Dental inspectors are constantly visiting camps and cantonments to inspect work done by the camp dentists in order to determine if the dental officers are competent, are correctly assigned, and to report on the general efficiency of the units. The same thorough care that is given to the men in this country is also given to the men in France. With each base, general or evacuation hospital there is attached a specialist in plastic surgery to correct deformities to face and jaws. Dental surgeons who have been specially trained in this class of work are also assigned to such hospitals.—*Army and Navy Register,* June 22d.

*Procaine and Novocaine Identical*

*To the Editor:* It appears that in certain quarters the attitude is taken that the local anesthetic sold as Procain is not identical with that marked as Novocaine. The sub-committee on Synthetic Drugs of the National Research Council believes it important that this misunderstanding should be corrected and hence offers the following explanation:

The monohydrochlorid of para-amino-benzoyl-diethyl-amino-ethanol, which was formerly made in Germany by the Farbwerke, vorm. Meister, Lucius and Bruening, Hoechst A. M., and sold under the trade-marked name "Novocaine," is now manufactured in the United States. Under the provisions of the Trading with the Enemy Act, the Federal Trade Commission has taken over the patent that gave monopoly for the manufacture and sale of the local anesthetic to the German corporation, and has issued licenses to American concerns for the manufacture of the product. This license makes it a condition that the product first introduced under the proprietary name "Novocaine" shall be called Procaine, and that it shall in every way be made the same as the article formerly obtained from Germany. To insure this identity with the German novocaine, the Federal Trade Commission has submitted the product of each firm licensed to the A. M. A. Chemical Laboratory to establish its chemical identity and purity, and to the Cornell pharmacologist, Dr. R. A. Hatcher, to determine that it was not unduly toxic.

In conclusion: Procaine is identical with the substance first introduced as Novocaine. In the interest of rational nomenclature, the first term should be used in prescriptions and scientific contributions.—Julius Stieglitz, Chicago, Chairman, Subcommittee on Synthetic Drugs, National Research Council, *Journal A. M. A.*, July 22, 1918.

*Difference Between Prophylactic Treatment and Oral Prophylaxis*

The general practitioner must also remember that the prevention of caries and periodontoclasia involves more than the giving of periodic prophylactic treatment. We make a distinction between oral prophylaxis and prophylactic treatment. We say that oral prophylaxis includes any agency which will prevent disease in the mouth. It consequently includes the prophylactic treatment, and in addition, the correction of malocclusion, building up of proper contacts, accurate finishing of fillings, hygienic construction of crowns and bridges, and many other things, failure in any one of which means the failure of the prophylactic treatment to accomplish its object.

When we consider the treatment of cases in which periodontoclasia has already developed, we come to a branch of dental practice which requires the development of an exacting technique and the possession of a certain kind of temperament. Success in this field can be attained without making it an exclusive specialty, but success can probably best be attained by the periodontist. The attitude of the profession to the problem of preventive treatment indicates to me a lack of real understanding of what oral prophylaxis means and also the relationship of this branch to the other departments of dental practice. Surely we should reach that conception of dental practice which will give the prophylactic treatment a position of equal importance with the placing of gold fillings and complicated prosthetic restorations, whatever means may be adopted for the actual performance of this operation. Naturally this means that the idea of the importance as well as the practicability must be inculcated into the minds of dental students at the very beginning of their course, before the mass and variety of reparative operations begin to bulk so large to them as to fill their entire horizon. As to those already in the profession, they may be reached through the journals, and perhaps best of all, by creating a demand for oral prophylaxis on the part of the public. This demand we are creating individually in our practices, but this of course reaches comparatively few. Would not occasional authorized articles in lay magazines be effective?—Dr. J. O. McCall, in *Dental Items of Interest.*

*Nerve Blocking Injections* I do not employ or advise the use of steel needles for deep nerve blocking injections nor do I use as few needles for all classes of local anesthesia as the essayist recommends. Personally, I employ platinum-iridium needles of known length and diameter for the routine injections, with the exception of blocking the terminal division of the infra-orbital nerve by the extra-oral method and for the initial injection prior to producing intra-osseous anesthesia. From personal experience I have found that I can do better work by employing several needles—say four or five—than to try to make all the various injections with one or two. When I say this number it includes the needles employed in the injections for extra-oral and intra-oral methods. This includes the various nerve blocking injections, terminal or infiltration, intra-osseous and the deep intra- and extra-oral injections. This, however, is due to personal experience and the operator who uses but two needles can probably do just as efficient work for certain injections, but when we consider all the methods of producing local anesthesia as I have already stated, it certainly requires more than two needles.

If one is not familiar with the anatomical parts, the relation of the bony-landmarks to the various nerve branches, arteries, veins, foraminae, he is not going to make the injections in an intelligent manner. Many dentists think all that is necessary in order to carry out this important phase of anesthesia is to have a hypodermic syringe, a needle and the injecting solution, but allow me to say, that a thorough knowledge of the subject is far more important than all the equipment put together. The operator who attempts to make the deep injections, without first familiarizing himself with the subject in all of its details, will no doubt encounter numerous failures.—Dr A. F. Smith, in *Journal N. D. A.*

*Sterilizing Anesthetic Solutions* According to the teachings of bacteriology we are aware of the fact that it is necessary to boil water for a period of fifteen minutes each day for three consecutive days in order to render the solution sterile. Distilled water does not mean sterile water, by any means, and it is always better to prepare the distilled water fresh every morning, than it is to keep a stock solution on hand for an indefinite period of time and take chances on injecting a solution which does not conform to the law of bacteriology. I wish to say a few words with reference to my findings with Ringer's solution. From the offices of ten Chicago operators I obtained samples of their stock Ringer solution, and took them to the bacteriological laboratory and made tests. Bouillon was inoculated with each sample, and after a forty-eight hour growth the specimens were transferred to petri dishes containing agar-agar; out of the ten specimens nine gave positive cultures. Out of ten specimens of stock distilled water, obtained in the same way, ten gave positive cultures. This should prove that stock distilled water and Ringer or Normal Saline solutions are by no means sterile. Many dentists do not boil their distilled water or Ringer solution, dissolving the tablets without taking any precautions whatsoever. In case sepsis follows such unscientific technic, they ought not to blame nerve blocking, but should place the blame upon themselves. When nerve blocking injections are skillfully made a very high percentage of cases will meet the approval of the most exacting operator. In order to obtain the best results the technic must be mastered and a strict adherence to dosage, isotonia and sterility of the injecting solution must be followed. And last but not least strict asepsis must be observed at all times.—Dr. A. F. Smith, in *Journal N. D. A.*

*Anesthetizing the Tonsils* The tonsils are completely anesthetized by blocking the palatine nerve branches which are given off Meckel's Ganglion in the spheno-maxillary fossa; and the tonsillar plexus, which is derived from branches from the glosso-pharyngeal, uniting with branches from the pharyngeal plexus. Two injections are necessary; one for the plexus and one for the palatine nerves. A straight needle with an extension hub is used for the plexus injection. The same needle is used with curved hub and extension for the palatine branches as employed for blocking the second division of the fifth nerve, on the posterior lateral surface of the superior maxillary bone. Complete anesthesia is produced within a few minutes following the injections and tonsillectomy is made a painless operation, and last but not least the operator has the co-operation of his patient and no inspiration of blood and mucous.—Dr. A. F. Smith, in *Journal N. D. A.*

*Capping Pulps* All carious dentin should be removed with spoon excavators, great care being observed not to remove that which is just over the pulp. Cotton, saturated with oil of cloves is then sealed in with one of the temporary cements. Two or three days later the temporary dressing is removed and the cap placed. A paste is made of oil of cloves and the powder of oxyphosphate cement. A metallic cap is then made, preferably of irridio-platinum. This cap is made hemispherical and concaved. It may be done with suitable contouring pliers, or by malleting with a round-end burnisher into a block of lead. The concaved cap is fitted to the bottom of the cavity and of such size and shape that its edges rest in sound dentin, thus bridging over and preventing future pressure upon the underlying pulp. The concaved hollow in the metallic cap is filled with the oil of cloves paste and placed over the point of suspected exposure (or, as in the first case cited, over the actual exposure) and is then protected with a layer of oxy-phosphate mixed to a creamy consistency and flowed over the capping. This provides the pulp with a metallic, bridge-like protection and the metallic cap likewise prevents the admixing of the oil of cloves paste with the superimposed oxyphosphate. It is deemed essential to thus maintain the hardening and sterilizing effect of the oil of cloves against the decalcified dentin left in place.

It should not be presumed that this method will be universally successful. Failure should be expected and provided for in every instance. It is therefore preferable to place temporary fillings over such caps for two or more months, before resorting to gold or amalgam. Of all fillings, the cast gold inlay is the most desirable in these cases, since it can be so constructed as to afford a ready means of access to the canal in case of death of the pulp, the possibility of which should never be overlooked. The wax pattern of the inlay having been made, a box should be cut in the underside immediately over the region of the pulp. In constructing this hollow, the pattern should be examined by transmitted light and should be carved until light readily passes through the wax. This will give an occlusal surface of wax so thin, that it can be readily pierced with a drill in case it should become necessary to enter for the removal of a dead pulp. To facilitate this, the box should be filled prior to cementation with a soft temporary stopping, so that after the drill should have pierced the gold, further approach to the pulp chamber will be easy, as it would not be, were cement permitted to enter this cavity.—Dr. Ottolengui, in *Around the Table.*

